# Growth Performance of Diminutive *Halophila ovalis* Seagrass Related to Substrate Condition in an Abandoned Mariculture Pond

**DOI:** 10.1002/ece3.73359

**Published:** 2026-04-05

**Authors:** Hongyi Wu, Shiman Li, Shunyang Chen, Liming Zuo, Ying Zhang, Jiahui Chen, Pengxiang Zheng, Guangcheng Chen

**Affiliations:** ^1^ Third Institute of Oceanography, Ministry of Natural Resources Xiamen China; ^2^ Observation and Research Station of Coastal Wetland Ecosystem in Beibu Gulf, Ministry of Natural Resources Beihai China; ^3^ Hebei Hydrological Engineering Geological Exploration Institute Shijiazhuang China; ^4^ School of Life Science and Technology Lingnan Normal University Zhanjiang Guangdong China

**Keywords:** biomass, cultivation, ecological restoration, seagrass, soil organic carbon

## Abstract

This study evaluated the feasibility of cultivating *Halophila ovalis* in an abandoned tidal‐connected mariculture pond and compared its growth performances under two substrate types, that is, raw pond soil and open sea soil. There was no significant difference in leaf number between the two substrate treatments during the first 2 months, while pond soil treatment showed a more rapid increase in the leaf number, with significantly higher values than those in the sea soil treatment from the 70^th^ day onward. However, carbon and nitrogen contents of seagrass biomass, as well as leaf chlorophyll *a* and *b*, were not affected by the substrate type. Soil organic carbon content increased in both treatments after the cultivation. The results suggested that *H. ovalis* could clonally reproduce in the tidal‐connected mariculture ponds, with a faster growth under the pond soil treatment. This highlights the potential use of these ponds as restoration sites to create seagrass communities and nursery environments to provide source plant materials for seagrass restorations.

## Graphical abstract

*Halophila ovalis* seagrasses clonally reproduce under both raw pond soil and open sea soil conditions in the tidal‐connected mariculture ponds. The pond soil supports a more rapid seagrass reproduction, as indicated by more leaf numbers of *H. ovalis* in the pond soil treatment from the 70th day onward.

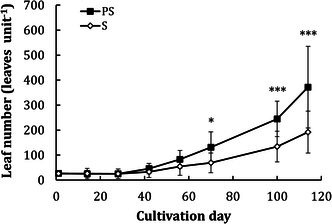

## 1 Introduction

Seagrasses are marine flowering plants that form large meadows in coastal waters, providing important ecosystem services, including supporting fisheries, facilitating nutrient cycling, and stabilizing sediments (Boudouresque et al. 2016; Nordlund et al. 2016). Moreover, seagrasses, along with mangroves and salt marshes, are blue carbon ecosystems that play crucial roles in carbon sequestration and climate change mitigation (Macreadie et al. 2021). However, seagrasses have undergone a global significant decline (Short et al. 2016; Mwikamba et al. 2024), with an accelerating rate reaching up to 7% per year since the 1990s (Waycott et al. 2009). This continued seagrass degradation has resulted in increasing efforts to restore seagrasses around the world (Van Katwijk et al. 2016).

The survival, growth and recruitment capacity of seagrass plants are influenced by various abiotic factors, for example, salinity, water temperature, light availability, water depth and substrate conditions (Rattanachot and Prathep 2015; Collier et al. 2016; Peralta et al. 2021). Substrate type is recognized as an important factor influencing the survival and growth of plants during seagrass transplantation or cultivation (Zabarte‐Maeztu et al. 2021). Although most seagrass species grow in sandy to muddy soils (Thorhaug et al. 2020), sandy substrates are often preferred for seagrass cultivation because their coarse particles enhance oxygen permeability, resulting in better survival and growth performance (Benham et al. 2019; Jiang et al. 2022). However, the sandy substrates are often nutrient‐poor, which would limit subsequent seagrass growth after transplantation, seagrasses are able to thrive under nutrient‐poor conditions (Zhang et al. 2021; Shen et al. 2023). A recent study by Valle et al. (2015) found that *Enhalus acoroides* growing in sandy substrates had higher shoot density than those growing in muddy substrates during the early stages of transplantation; however, the long‐term survival rates of the transplanted units were higher in muddy substrates. Other studies also found that *Zostera marina* and *E. acoroides* growing in soils with medium to high silt and clay contents had better growth and establishment than those in sandy soils, owing to the higher nutrient content in the former (Lanuru 2011; Zhang et al. 2015).

Anthropogenic activities, such as mariculture and salt production, have been increasing in coastal regions over the past few decades and have caused extensive losses of coastal wetlands (Jayanthi et al. 2018; Stiller et al. 2019; Luo et al. 2022), resulting in the close proximity of coastal wetlands to these mariculture or salt ponds. Studies have shown that some mariculture and salt ponds provide habitats for seagrass distributions (Triest and Sierens 2009; Yu and Den Hartog 2014; De Los Santos et al. 2020). For example, *Ruppia* spp. are widely found in ponds in China and the Mediterranean (Triest and Sierens 2009; Yu and Den Hartog 2014), and in Ria Formosa, South Portugal, *Cymodocea nodosa* and *Zostera* spp. have been observed to form dense coverage in tidal‐connected reservoir ponds which provide natural seawater for surrounding mariculture ponds (De Los Santos et al. 2020). In South China, seagrasses have been observed in tidal‐connected reservoir ponds and their associated abandoned mariculture ponds were found, which were vegetated by species including *Ruppia* spp. and *Halophila ovalis* (R.Brown) J.D.Hooker, 1858 (personal observation). Given that pond disuse has become common due to unproductive culture, disease, or the increasing need for the conversion of ponds back to coastal wetlands for sustainable development purposes (Stevenson et al. 1999; Tian et al. 2024), the abandoned mariculture ponds provide potential space for seagrass cultivation and restoration, as also proposed by other studies (Van Katwijk et al. 2021; Adhavan et al. 2022).

The cultivation of seagrass seedlings has been widely applied in seagrass restoration projects to provide source plants (Gamble et al. 2021), which usually involves aquaria systems with controlled environments optimal for the seagrass growth (Tanner and Parham 2010; Van Katwijk et al. 2021; Zhang et al. 2024). However, after transplantation of the cultivated seagrasses into the sea area, changes in environment would likely limit the growth performance and even cause mortality of plants, reducing the restoration effectiveness (Zhang et al. 2024). Recent studies have implied the feasibility of in situ growing of seagrass seedlings or shoots in the natural sea area to provide the plant materials for seagrass restorations (e.g., Mancini et al. 2024; Pansini et al. 2025). These suggests that the abandoned mariculture ponds, due to tidal connectivity and substrate diversity, may provide suitable environment conditions similar to the natural ones for seagrass cultivations; however, this remains to be proved.

*H. ovalis* is the most widely distributed tropical seagrass species in the Indian and Pacific Oceans, particular in southern and southeastern Asia (Short et al. 2007), and occurs from intertidal to shallow subtidal habitats with a broad substrate adaptability, from coarse sands in open marine environments to clay‐rich sediments in sheltered waters (Tanaka and Kayanne 2007; Herbeck et al. 2014; Kaewsrikhaw and Prathep 2014). *H. ovalis* is listed as Least Concern on the International Union for Conservation of Nature Red List (Short et al. 2010), while it's undergoing habitat loss or degradation due to anthropogenic disturbances and climate changes across the region (Yaakub et al. 2014; Jiang et al. 2020; Lin et al. 2020). In China, *H. ovalis* mainly occurs in Hainan Province and Beibu Gulf (Zhou et al. 2023), and frequent and unregulated anthropogenic disturbances, such as mariculture and fishing activities, have resulted in substantial declines in the area and shoot densities of the *H. ovalis* meadows (Jiang et al. 2020; Liu et al. 2025). Nevertheless, studies on the cultivation and restoration of *H. ovalis* are limited compared with those large species in China.

Seagrasses reproduce both sexually and asexually. Their clonal reproduction via rhizome growth typically controls seagrass productivity and plays a key role in the development and maintenance of seagrass meadows (Marbà and Duarte 1998; Nakaoka and Aioi 1999). *H. ovalis* has thin, long‐internoded, fast‐branching rhizomes and can form a spiral rhizome network with low spread efficiency and high space‐filling capacity, enabling fast elongation and spread of rhizomes to rapidly recruit shoots (Marbà and Duarte 1998). These fast growth strategies of *H. ovalis* enable its cultivation based on clonal reproduction. In this study, the *H. ovalis* growth was measured in a tidal‐connected abandoned mariculture pond in Beihai City, China, to examine the feasibility of cultivating *H. ovalis* by clonal reproduction in the pond as a nursery purpose. The growth performance was also compared between the seagrasses cultivated under two substrate types, such as raw pond soil and open sea soil. We hypothesize that *H. ovalis* will clonally reproduce in the pond and show better growth performance under raw pond soil condition due to its higher nutrient availability compared to open sea soil.

## 2 Materials and Methods

### 2.1 Study Area

**FIGURE 1:**
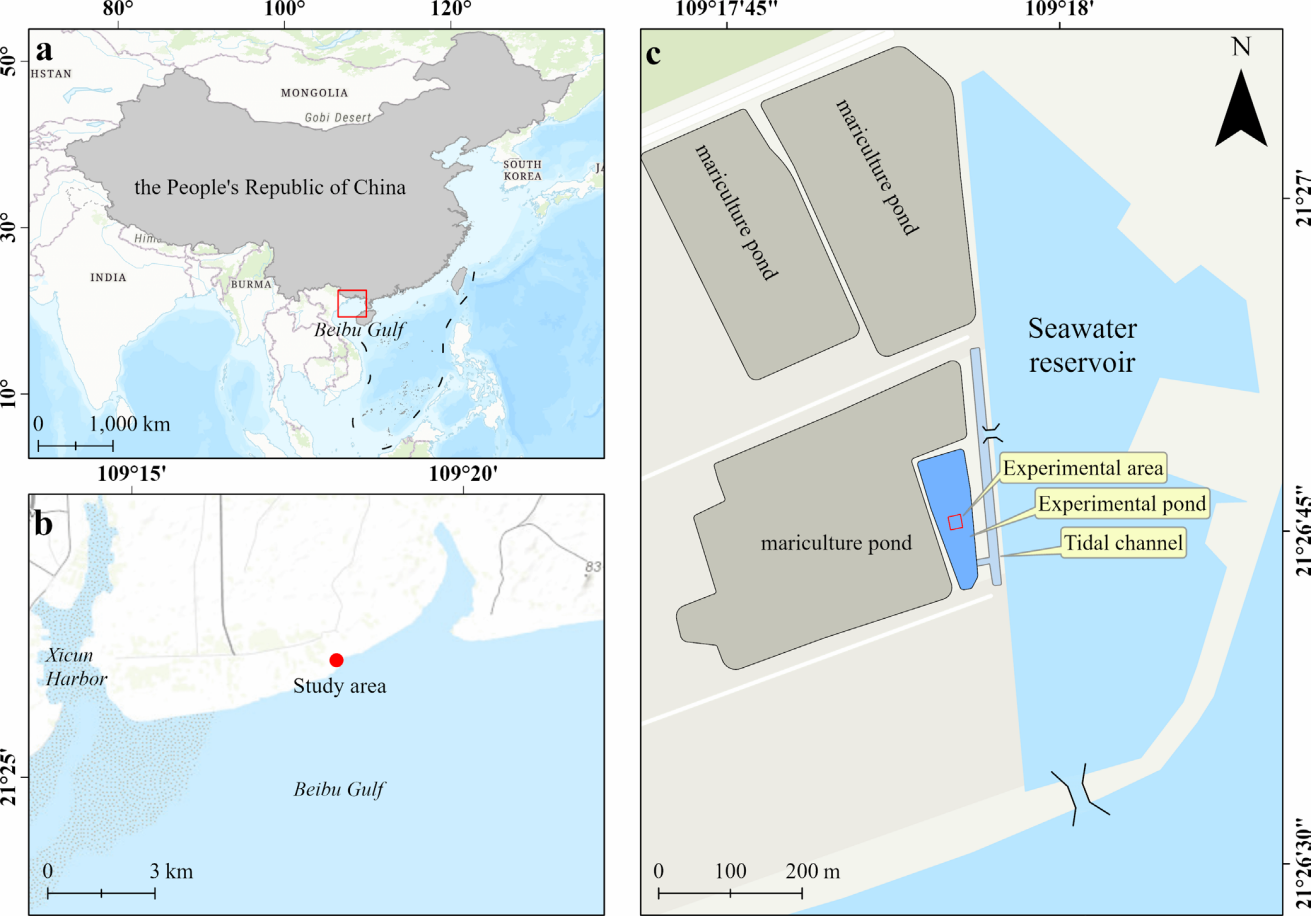
Location of the Beibu Gulf and study area (a), location of the study site along the Beibu Gulf coast (b), and the abandoned mariculture pond used for seagrass cultivation (c), in Beihai City, China. The map was created using ArcGIS Pro version 3.0 (https://www.esri.com). Administrative boundary data for China were obtained from the National Platform for Common Geospatial Information Services (https://www.tianditu.gov.cn/). The base maps in (b) and (c) were derived from the World Topographic Map (http://services.arcgisonline.com/arcgis/rest/services) and the digitized Google Earth imagery (Version 7.3, https://www.google.com/earth/) (Image 2021 DigitalGlobe).

The cultivation experiment was conducted in a tide‐connected mariculture pond in Beihai City, Guangxi Province, South China (Figure 1). The study area has a subtropical monsoon climate, and the monthly mean temperature ranges from 15.0°C in January to > 28.0°C (from June to August); the annual precipitation is 1573 mm, with > 80% of the annual precipitation occurring in the wet season (China Gulf Chronicles Compilation Committee 1993; Zheng et al. 2023). The study area experiences an irregular diurnal tidal cycle, with an annual tidal range of 2.53 m, and the seawater salinity varies between 26 and 31 throughout the year. Five seagrass species, such as *Zostera japonica* , *Syringodium isoetifolium* , *Halodule uninervis* , *Halophila beccarii*, and *H. ovalis* , have been recorded in the shallow waters along the coast of Guangxi (Shi et al. 2010).

The mariculture pond was abandoned around 2020, and the pond dike was damaged, allowing the pond to connect directly with a seawater reservoir. The seawater reservoir with an area of 24 ha connects to the sea through a tidal gate. The abandoned pond had an area of ~0.3 ha, and the water depth in the pond fluctuated with the tide, with a water depth > 20 cm during low‐tide periods. *H. ovalis* was widely found in the reservoirs, tidal channels and abandoned ponds and had formed vegetation patches in the pond. The sea soil was collected from an adjacent bare flat area in the open sea, located near an existing natural *H. ovalis* ‐dominated meadow, namely Zhulin seagrass meadow. The Zhulin meadow is subjected to intensive fishing stress, and fishing by digging or using high‐pressure water jets for clam and sandworm ( *Sipunculus nudus* ) has resulted in the decline in seagrass extent and reduced coverage and shoot density (Su et al. 2017).

### 2.2 Experimental Design

Seagrass sods with an area of 78.5 cm^2^ (i.d. 10 cm) were collected in situ inside the pond, by gently turning PVC tubes to a depth of 10 cm in February. A total of 19 sods were collected, three of which were randomly collected for measurements of the background characteristics of the seagrass samples, including above‐ and below‐ground biomass, Chlorophyll *a* and Chlorophyll *b* contents in the leaves. For the remaining sods, each was transplanted into a plastic basket with dimensions of 32 cm × 24 cm × 9 cm (length × width × height), and substrate was then filled into the basket to form an experimental unit. These permeable containers support the survival and growth of seagrass shoots after cutting, and are also used in other seagrass cultivation studies (Zhao et al. 2023; Zhang et al. 2024). The inner side of the basket was covered by a nylon mesh to prevent the loss of substrate. The mesh size (3.35 mm) was greater than the rhizome diameter of *H. ovalis* plants (< 2 mm) of a local community in Beihai (Xu et al. 2011), allowing the rhizome and root penetration through the side of the baskets. Two substrate types were applied in this study, that is, raw pond soil and the sea soil, and 8 experimental units were set up for each. The sandy and silty contents of the raw pond soil were 88% and 8.3%, respectively, whereas the sea soil contained 98.5% sandy components. The substrate textures fall within the natural substrate spectrum known for this species (Tanaka and Kayanne 2007).

The experimental units were randomly placed within the central pond area under a similar water depth condition and remained flooded during the low‐tide period. The experimental units were conditioned in the pond for 1 day, after which three replicate units were randomly collected from each treatment for soil characteristic analysis. The leaf numbers were counted approximately every 2 weeks (except on the 84^th^ day) during the experiment, which ended on the 114^th^ day when the plants were observed to extend to the margin of the basket in some units without apparent root and rhizome penetration through the side of the baskets.

### 2.3 Sample Analysis

In the last measurement campaign in June, three replicate units of each treatment were randomly selected for biomass measurement, and the seagrass plant and soil samples were also collected for subsequent analysis. For each replicate unit, the leaf length and width of three shoots were measured. The aboveground and belowground biomasses were sampled and weighed after drying at 60°C for 48 h. The chlorophyll was extracted from fresh leaf segments using 5 mL of N‐dimethylformamide reagent, and the absorption was measured spectrophotometrically to determine the chlorophyll *a* and *b* contents (Xiao and Wang 2005). The ammonia (NH_4_^+^‐N) content in the fresh soil samples was determined following Chen et al. (2020). The organic carbon (OC) and total nitrogen (TN) contents in the plant and soil samples were analyzed via a TOC analyzer coupled with a nitrogen analyzer (Vario TOC Cube, Elementar Analysensysteme, Germany), and the soil samples were acidified with diluted HCl (5%) and then oven dried at 40°C to remove carbonates before analysis. The total phosphorus (TP) content was determined via a continuous flow analyzer (Futura II, Alliance Instruments, France) following digestion.

### 2.4 Statistical Analysis

Mean and standard deviation of the parameters were calculated. The homogeneity of variance and normality of the variables were examined via the Shapiro–Wilk and Levene tests. When necessary, the data were transformed with the Blom method to normality and homogeneity (Blom 1958; Soloman and Sawilowsky 2009). The effects of cultivation time and substrate types on leaf number were tested using two‐way analysis of variance (ANOVA). The same test was used to examine the effects of sampling time (initial vs. final sampling) and substrate types on the soil OC and nutrient contents. For plant nutrient contents, their differences between biomass type (above‐ vs. below‐ground biomass) and substrate types were tested using two‐way ANOVA. When the difference was significant at *p* < 0.05, a Tukey Post Hoc test was applied to determine where the differences lay. The differences in biomass, above‐to‐belowground biomass ratio, leaf chlorophyll *a* and *b* contents among the background group and the two substrate treatments (measured at the end of the experiment) were tested using one‐way ANOVA. The leaf morphological features were tested using an independent sample *t*‐test. All data analyses were conducted in IBM SPSS Statistics (version 22.0, Chicago, IL, United States).

## 3 Results

### 3.1 Vegetation Characteristics

**FIGURE 2:**
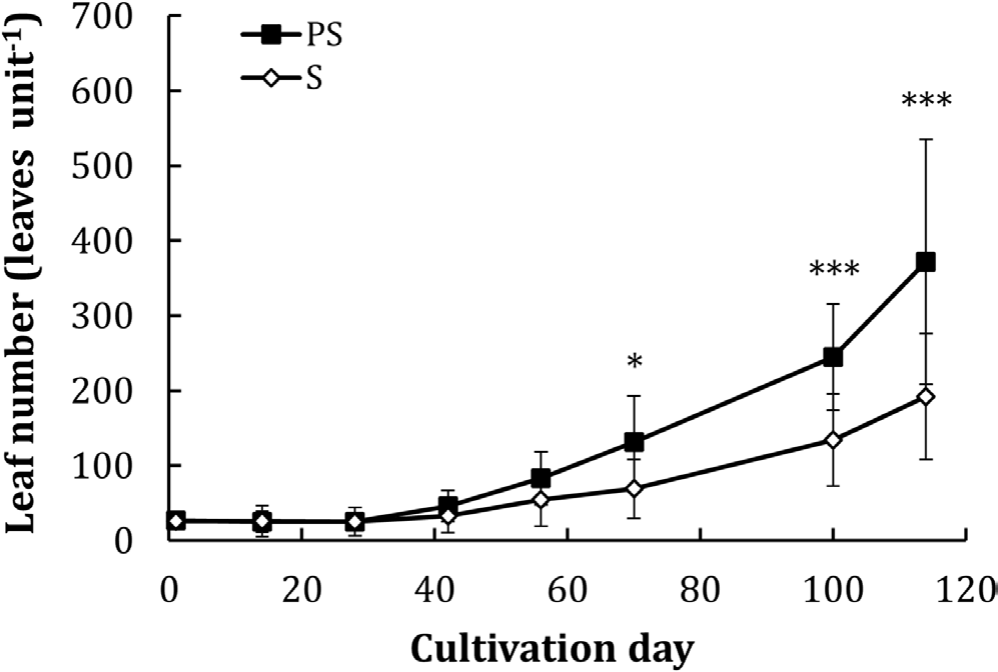
Changes in leaf number with cultivation time in the two substrate treatments. The means and standard deviations of eight replicates are shown. PS, Pond soil; S, Sea soil. At each time point, * indicates *p* < 0.05 and *** indicates *p* < 0.001.

Leaf number was comparable between the two treatments at the beginning of the experiment, that is, 27 ± 12 and 26 ± 11 leaves per unit for treatment PS and S, respectively (Figure 2). Leaf number changed with cultivation time (*F* = 43.27, *p* < 0.001) and substrate treatment (*F* = 23.76, *p* < 0.001), but the temporal changes were related to the substrate type, as indicated by a significant interaction between cultivation time and substrate type (*F* = 5.23, *p* < 0.05). There was no significant change in leaf number in the first month in the raw pond soil treatment (PS), and a significant increase in leaf number was measured since the 56^th^ day (Table S1). For the sea soil (S) treatment, there was no leaf number increase until the 100^th^ day (Table S1). There was no significant difference in leaf number between the two treatments in the first 2 months, while the treatment PS showed a faster increase from the 70^th^ day onward and significantly greater leaf numbers than those of treatment S (Table S2), with leaf number reaching 372 ± 163 and 192 ± 84 leaves per unit in treatments PS and S at the end of the experiment, respectively (Figure 2).

The seagrass had a background biomass of 0.33 ± 0.01 g DW per unit, with an above‐ to belowground biomass ratio of 1.4 (Table 1). At the end of the experiment, there were no significant differences in either biomass or above‐ and below‐ground biomass ratio (*p* > 0.05) between the two treatments. The leaf chlorophyll *a* and *b* contents were 0.59 ± 0.06 and 0.38 ± 0.05 mg g^−1^ at the beginning of this experiment, and their contents were comparable between the treatments at the end of the experiment (*p* > 0.05). Differences in the morphological features of seagrass leaves were found, with greater leaf length measured in the treatment PS than the treatment S (*p* < 0.05).

**TABLE 1:** Characteristics of seagrass biomass, leaf morphology, and chlorophyll content.

Parameters	Background	Treatment PS	Treatment S
Biomass (g)	0.33 ± 0.01	1.57 ± 1.20	0.65 ± 0.38
Above‐ to below‐ground biomass ratio	1.4 ± 0.6	1.9 ± 0.7	1.5 ± 0.4
Chlorophyll *a* (mg g^−1^)	0.59 ± 0.06	0.82 ± 0.37	0.98 ± 0.15
Chlorophyll *b* (mg g^−1^)	0.38 ± 0.05	0.51 ± 0.29	0.63 ± 0.16
Leaf length (mm)	—	14.00 ± 2.46	12.43 ± 2.12
Leaf width (mm)	—	5.18 ± 1.42	4.79 ± 1.23

*Note:* The means and standard deviations of three replicates are shown.

Abbreviations: PS, pond soil; S, sea soil.

**FIGURE 3:**
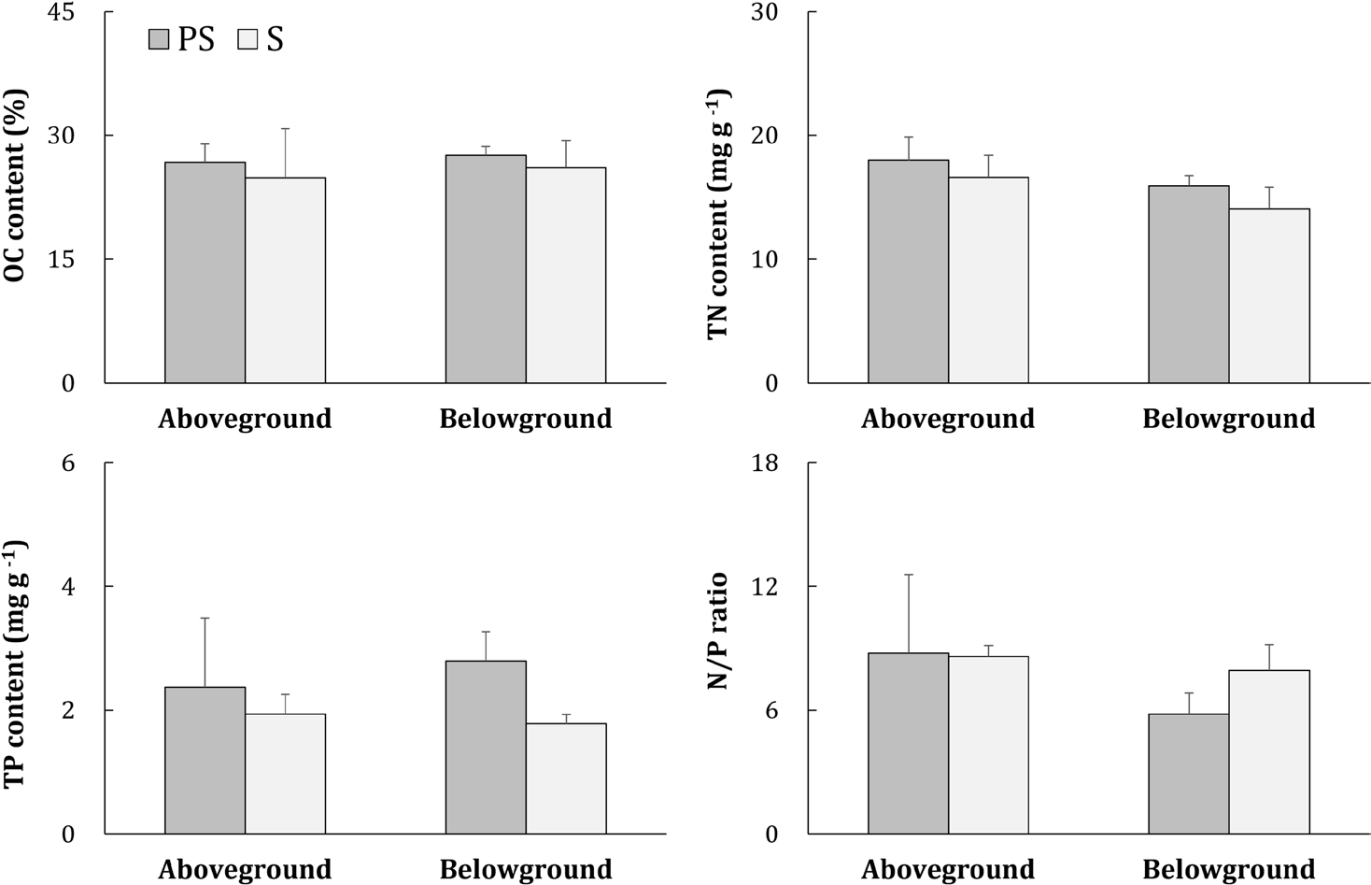
Nutrient contents of aboveground and belowground biomass in the two substrate treatments. The means and standard deviations of three replicates are shown. Same abbreviations as Figure 2.

The OC, TN and TP contents in the seagrass samples were 26.7% ± 0.6%, 17.3 ± 1.7 mg N g^−1^ and 2.2 ± 0.3 mg P g^−1^, respectively, on the 1st day. After cultivation, the OC content of *H. ovalis* (24.9%–27.6%) was comparable in aboveground biomass and belowground biomass and was not affected by the substrate (Table 2). Similar to OC, there were no significant differences in TN and TP between the two substrate treatments. However, the TN content was greater in the aboveground biomass than in the belowground biomass (Figure 3). The two treatments had similar TP contents in the above‐ and belowground biomass. In terms of the N/P ratio, comparable values were observed in both treatments as well as in above‐ and belowground biomass.

**TABLE 2:** *F* values of two‐way ANOVA tests showing the effects of biomass type (above‐ vs. below‐ground) and substrate type on elements in seagrass.

Parameters	Biomass type	Substrate type	Interaction
OC content	0.24	0.65	0.01
TN content	5.97*	3.00	0.07
TP content	0.13	3.84	0.62
N/P ratio	2.34	0.66	0.91

* Significant effect at *p* < 0.05.

### 3.2 Soil Chemical Parameters

**FIGURE 4:**
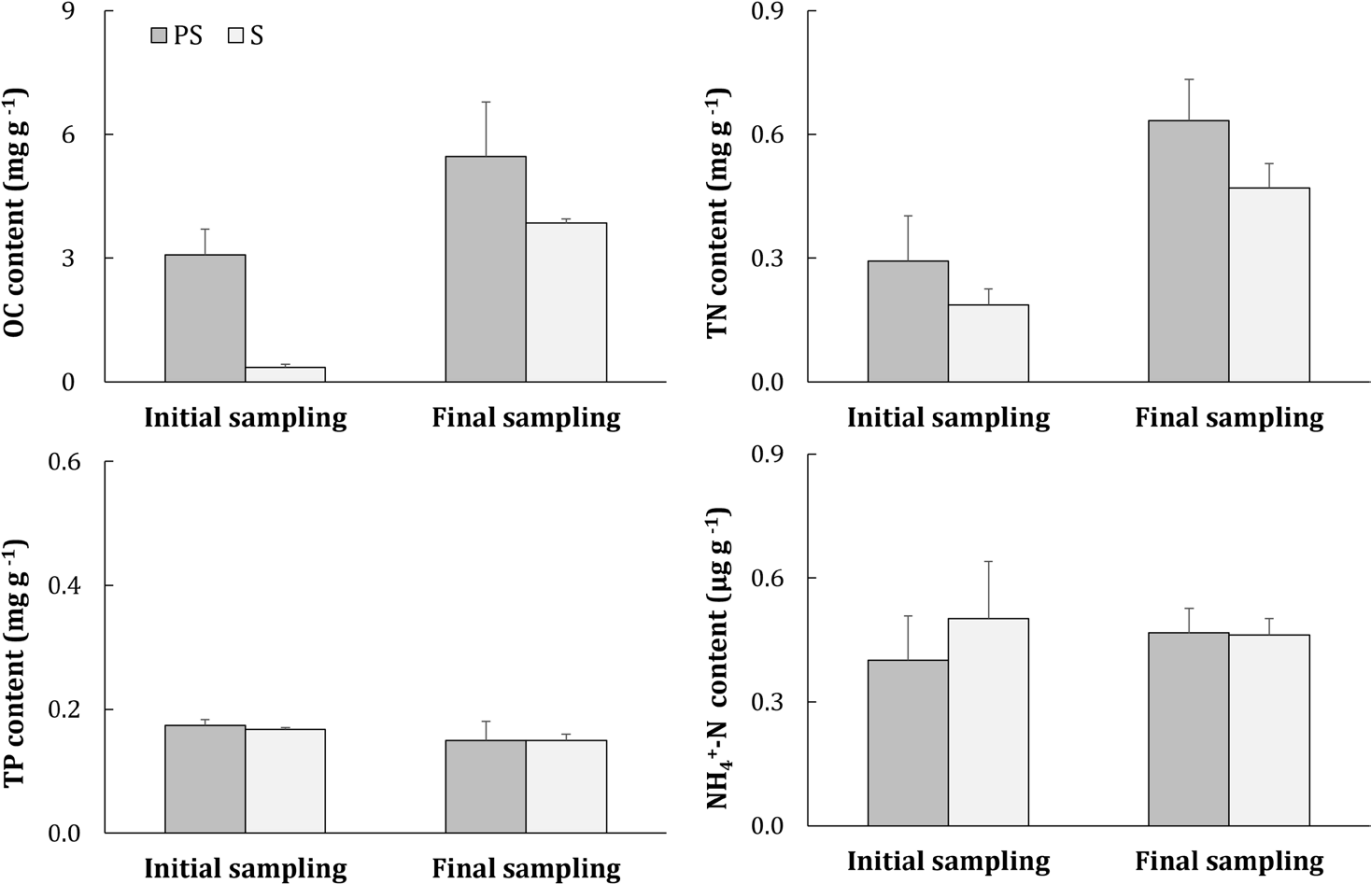
Soil OC and nutrient contents in the two substrate treatments at initial and final samplings. The means and standard deviations of three replicates are shown. Same abbreviations as Figure 2.

Cultivation time and substrate type had significant impacts on soil OC and TN contents (Table 3). Significant increases in soil OC were found in the PS and treatment S at the end of the experiment (Figure 4). The soil TN content was greater in the treatment PS than in the treatment S and was greater at the end of the experiment (114^th^ day) than on the 1st day for the two substrate treatments. The soil TP and NH_4_^+^‐N contents were similar between the two treatments in both measurement campaigns.

**TABLE 3:** *F* values of two‐way ANOVA tests showing the effects of sampling time (initial vs. final samplings) and substrate type on soil parameters.

Parameters	Cultivation time	Substrate type	Interaction
Soil OC content	48.27***	26.42***	1.76
Soil TN content	42.85***	8.04**	0.35
Soil TP content	3.26	0.05	0.14
Soil NH_4_^+^‐N content	0.06	0.79	0.94

** Significant effects at *p* < 0.01.

*** Significant effects at *p* < 0.001.

## 4 Discussion

In this study, the growth performance of *H. ovalis* under the raw pond soil and open sea soil was investigated, and the seagrass survived and showed clonal reproduction within the experimental unit under both substrate types in the tidal‐connected pond. The cultivated seagrasses showed apparent increases in leaf numbers since approximately 2 months following cultivation, that is, in April. This coincided with the growth phenology of *H. ovalis* , which has the highest canopy coverage, shoot density and biomass in spring in Beihai (Zheng et al. 2026). Wirachwong and Holmer (2010) also found that the *H. ovalis* started to increase its shoot density in April or May in Trang Province, Thailand. The area of the cultivation unit was 736 cm^2^, and the seagrass biomass in the unit was estimated as 8.83–21.33 gDW m^−2^ at the end of this experiment, falling within the respective range of *H. ovalis* (17.38–37.68 gDW m^−2^) reported in the local *H. ovalis* communities under natural conditions in Beihai (Xu et al. 2011; Zheng et al. 2026), and the ultimate leaf density (2608–5258 leaves per m^−2^) in our experimental units was even higher than other *H. ovalis* communities (250–1320 leaves per m^−2^) under natural conditions (Xu et al. 2011; Kaewsrikhaw et al. 2016; Adhavan et al. 2022). The results suggest that the mariculture ponds with tidal connections are suitable sites for *H. ovalis* conservation and could be used for seedling cultivation for seagrass restoration. The seagrass vegetated ponds also provide alternative donor sources for seagrass cultivation to mitigate the collecting impacts on natural meadows. In this study, the basket‐based experimental units were used and placed on the surface of the pond sediment, which provided a different environmental condition for *H. ovalis* plants and would lead to a subsequent different growth performance from that observed under natural conditions. This further implies future studies on the seagrass growth performance and restoration effectiveness following the transplantation of sods to the natural open sea (e.g., the Zhulin meadow) for seagrass restoration.

There was a clear difference in the growth performance of seagrass between the two substrate types in terms of the leaf number, with seagrass in the treatment PS producing more leaves, which supports the hypothesis that pond‐soil cultivation promotes seagrass growth by clonal reproduction. This may be owing to the slightly higher proportion of clay and silt in treatment PS than in treatment S, which supports better growth performance, as demonstrated by numerous previous studies (Nishijima et al. 2015; Valle et al. 2015; Størdal et al. 2023). It has been suggested that the above‐ground biomass and growth rate of seagrass are positively correlated with the silt and clay contents in the soil (Zhang et al. 2015; Jiang et al. 2020). Moreover, the clay and silt components fill the gaps between larger particles and thereby could increase the degree of root anchoring of plants within the soil (Nishijima et al. 2015), which further supports better seagrass growth performance.

This study found high intra‐treatment variability in leaf number in both treatments at the end of the experiment. This might be partially attributed to the variabilities in leaf number of those raw sod materials in the two treatments. Moreover, morphometric traits including the shoot density and leaf traits of seagrass plants are highly dependent on local environmental conditions and show interspecific heterogeneity even within a certain area (Gorman et al. 2016; Ambo‐Rappe et al. 2025). In this study, a seagrass cultivation experiment was done within the pond area and the experimental units were placed closely and randomly on top of the pond sediment under a similar environmental condition. However, the micro‐scaled geomorphological and hydrological heterogeneities among the units may exist within the experimental area, which would further result in the intra‐treatment variability of seagrass growth. Such large variations in shoot density and biomass of *H. ovalis* were also found in field sites (Ambo‐Rappe et al. 2025; Zheng et al. 2026) or indoor aquaria systems (Van Barneveld Pérez and Samper‐Villarreal 2025). Nevertheless, this intra‐treatment variability would not interfere with the finding that the pond soils support a better seagrass reproduction because significantly higher leaf number was found in the treatment PS at the end of the experiment.

Compared with treatment S, the seagrass in treatment PS presented longer leaves, a characteristic commonly associated with greater nutrient availability (Jiang et al. 2019; Thomsen et al. 2022). The higher mean biomass in treatment PS and close biomass TN contents between the two treatments suggested greater nitrogen assimilation in treatment PS. The increased NH_4_^+^‐N uptake by plants requires additional carbon for their assimilation of NH_4_^+^‐N into amino acids, and this increased carbon demand is primarily met through photosynthetic carbon fixation in leaf tissues (Turpin et al. 1990; Huppe and Turpin 1994), leading to larger leaves of seagrass under nitrogen enrichment (Lee and Dunton 2000). Leaf uptake of inorganic nitrogen could be another important nitrogen uptake pathway besides the soil NH_4_^+^‐N utilization when the seagrasses are exposed to inorganic nitrogen enrichment in seawater (Iizumi and Hattori 1982; Touchette and Burkholder 2000). The seawater surrounding our study area was suggested to be nutrient enriched because of inputs from agricultural runoff, mariculture wastewater, and domestic sewage (Ouyang et al. 2022), which would allow the intake of more nitrogen by seagrass leaves in treatment PS as a supplementary source of their nitrogen demand. There was no significant difference in chlorophyll content between the two treatments at the end of the experiment. Previous studies have demonstrated that the leaf chlorophyll level is more related to light availability and water depth than to nutrient level (Wagey 2014; Ravaglioli et al. 2017), and the experimental units in this study were cultivated at similar water depths, resulting in a similar chlorophyll level in the two treatments.

Despite the higher soil TN content in treatment PS than in treatment S, the TN content in the seagrass was similar between these two substrate treatments in both their above and below‐ground biomass. Diminutive seagrasses have a low nitrogen demand even under high nitrogen availability and could regulate their nutrients assimilation to maintain their growth (Viana et al. 2019, 2020). Under nutrient‐deficient conditions, plants usually allocate more biomass to belowground tissues to increase the surface area for nutrient absorption (Gleeson 1993; Vogt et al. 1993). *H. ovalis* could produce longer roots and a higher proportion of belowground biomass under nutrient‐limited condition than nutrient rich condition (Jiang et al. 2019; Song et al. 2021). Although no significant differences were observed in the above‐ to belowground biomass ratios between the treatments, the treatment PS had a higher average ratio than the treatment S, indicating more belowground biomass allocation in treatment S. This strategy might allow the seagrasses in the treatment S to take up adequate TN from the substrate to maintain a TN level similar to that of treatment PS. Such an allocation pattern of biomass was also found for seagrass to adapt to other environmental changes (Zhang et al. 2021). Pansini et al. (2025) found a more extended root length of *Posidonia oceanica* seedlings originating from the warm coast than those from the colder coast under a warm cultivation condition, with such an origin‐associated difference less apparent under a colder cultivation condition; while the origin‐associated difference in leaf length extension showed a similar pattern under the two thermal regimes.

In this study, increases in soil OC and TN contents were detected in both treatments after a 114‐day cultivation, further demonstrating that seagrass colonization could enhance the soil OC/TN contents, an important restoration outcome as demonstrated by numerous previous studies (Greiner et al. 2013; Lange et al. 2022; Liu et al. 2023). The increase in soil OC (~3 mgC g^−1^) was greater than the accumulation rates reported in some other seagrass restorations in natural environments (Liu et al. 2023; Rahayu et al. 2023). For instance, Greiner et al. (2013) reported an increase from 3.6 to 5.2 mg g^−1^ in top 10 cm soil 10 years after restoration in South Bay on the Eastern Shore of Virginia, USA, and the surface soil (3 cm) showed an accumulation of 0.21 mg C g^−1^ in a 2‐year‐old *T. hemprichii* meadow in Li'an Bay in Hainan, South China (Liu et al. 2023). The apparent increase in soil OC in this study was likely due to the sheltered environment in the pond reducing the export of seagrass‐derived material by tides and increasing the accumulation of organic matter. Previous studies have suggested that seagrasses growing in sheltered environments (i.e., lagoon areas) with reduced hydrodynamic forces tend to exhibit increased carbon sequestration rates than those growing in open areas (Jankowska et al. 2016). The present study was based on an experimental short‐term cultivation of *H. ovalis* in the pond, and the results suggest that abandoned mariculture ponds provide not only feasible space for the seagrass cultivation but also substantial carbon sequestration benefits from seagrass restoration within the pond areas. Regarding that the soil OC accumulation following seagrass colonization is influenced by several factors, including species, duration of restoration, and site conditions (Gullström et al. 2018; Lei et al. 2023; Rahayu et al. 2023), the carbon sequestration potential of seagrass restoration in mariculture ponds associated with various factors such as seagrass species and restoration time deserve further studies.

## Author Contributions

**Hongyi Wu:** formal analysis (lead), writing – original draft (lead), writing – review and editing (lead). **Shiman Li:** formal analysis (equal), investigation (lead), methodology (equal). **Shunyang Chen:** conceptualization (supporting), investigation (lead). **Liming Zuo:** supervision (equal), visualization (equal). **Ying Zhang:** validation (equal), visualization (equal). **Jiahui Chen:** investigation (equal). **Pengxiang Zheng:** investigation (equal). **Guangcheng Chen:** conceptualization (lead), methodology (lead), resources (equal), writing – review and editing (lead).

## Funding

This work was supported by the Science & Technology Fundamental Resources Investigation Program (2023FY100804) and the Scientific Research Foundation of the Third Institute of Oceanography, MNR (2020017). The Ant Foundation also contributed.

## Conflicts of Interest

The authors declare no conflicts of interest.

## Supporting information


**Table S1:** Multiple comparisons of leaf number among cultivation time within each substrate type treatment based on Tukey Post Hoc test.
**Table S2:** Comparisons of the effects of substrate type on leaf number at each cultivation time based on Tukey Post Hoc test.
**Table S3:** Leaf number (leaves per experimental unit, mean ± SD, *n* = 8) at different cultivation times in the two substrate treatments.
**Table S4:** Nutrient contents (mean ± SD, *n* = 3) of aboveground and belowground biomass in the two substrate treatments.
**Table S5:** Soil OC and nutrient contents (mean ± SD) at initial and final sampling in the two substrate treatments (*n* = 3).

## Data Availability

The datasets used during the current study are available in the Supporting Information (Tables S3–S5).
