# Supplementary material for: Growth Performance of Diminutive Halophila ovalis Seagrass Related to Substrate Condition in an Abandoned Mariculture Pond
Source: Ecol Evol. 2026 Apr 5;16(4):e73359. doi: 10.1002/ece3.73359 (PMC13052325; doi:10.1002/ece3.73359)
Supplement: Supplementary file 1 — Table S1: Multiple comparisons of leaf number among cultivation time within each substrate type treatment based on Tukey Post Hoc test. Table S2: Comparisons of the effects of substrate type on leaf number at each cultivation time based on Tukey Post Hoc test. Table S3: Leaf number (leaves per experimental unit, mean ± SD, n = 8) at different cultivation times in the two substrate treatments. Table S4: Nutrient contents (mean ± SD, n = 3) of aboveground and belowground biomass in the two substrate treatments. Table S5: Soil OC and nutrient contents (mean ± SD) at initial and final sampling in the two substrate treatments (n = 3). [file ECE3-16-e73359-s001.docx]

**Supplementary data**

**Growth performance of diminutive *Halophila ovalis* seagrass related to substrate condition in an abandoned mariculture pond**

Hongyi Wu^1^, Shiman Li^2^, Shunyang Chen^1,2^, Liming Zuo^3^, Ying Zhang^4^, Jiahui Chen^1,2^, Pengxiang Zheng^1^, Guangcheng Chen^1,2^

^1^ Third Institute of Oceanography, Ministry of Natural Resources, Xiamen, China

^2^ Observation and Research Station of Coastal Wetland Ecosystem in Beibu Gulf, Ministry of Natural Resources, Beihai, China

^3^ Hebei Hydrological Engineering Geological Exploration Institute, Shijiazhuang, China

^4^ School of Life Science and Technology, Lingnan Normal University, Zhanjiang, Guangdong, China

Table S1 Multiple comparisons of leaf number among cultivation time within each substrate type based on Tukey Post-Hoc test.

| Cultivation time | Leaf number | |
| --- | --- | --- |
|  | Pond soil | Sea soil |
| 1 | ab | a |
| 14 | a | a |
| 28 | a | a |
| 42 | ab | a |
| 56 | bc | a |
| 70 | c | a |
| 100 | d | b |
| 114 | e | c |

Different letters within each column indicate significant differences among the cultivation time within the same substrate type (Tukey Post-Hoc test, p < 0.05) .

Table S2 Comparisons of the effects of substrate type on leaf number at each cultivation time based on Tukey Post-Hoc test.

| Cultivation time | Leaf number |
| --- | --- |
| 1 | Pond soil = Sand (p > 0.05) |
| 14 | Pond soil = Sand (p > 0.05) |
| 28 | Pond soil = Sand (p > 0.05) |
| 42 | Pond soil = Sand (p > 0.05) |
| 56 | Pond soil = Sand (p > 0.05) |
| 70 | Pond soil > Sand (p < 0.05) |
| 100 | Pond soil > Sand (p < 0.001) |
| 114 | Pond soil > Sand (p < 0.001) |

Table S3. Leaf number (leaves per experimental unit, mean ± SD, n = 8) at different cultivation times in the two substrate treatments.

| Cultivation time (day) | Pond soil treatment | Sea soil treatment |
| --- | --- | --- |
| 1 | 26.88 ± 11.54 | 26.38 ± 11.46 |
| 14 | 25.12 ± 13.68 | 26.00 ± 20.54 |
| 28 | 25.12 ± 10.92 | 24.88 ± 18.98 |
| 42 | 46.12 ± 20.90 | 32.62 ± 22.16 |
| 56 | 82.88 ± 35.45 | 54.62 ± 35.17 |
| 70 | 131.75 ± 60.86 | 69.00 ± 39.48 |
| 100 | 245.00 ± 70.87 | 134.25 ± 61.16 |
| 114 | 372.00 ± 163.26 | 192.12 ± 84.10 |

Table S4 Nutrient contents (mean ± SD, n = 3) of aboveground and belowground biomass in the two substrate treatments.

| Parameters | Aboveground biomass | | Belowground biomass | |
| --- | --- | --- | --- | --- |
|  | Treatment PS | Treatment S | Treatment PS | Treatment S |
| OC content (%) | 26.76 ± 2.22 | 24.86 ± 5.96 | 27.58 ± 1.06 | 26.09 ± 3.32 |
| TN content  (mg g⁻¹) | 17.99 ± 1.90 | 16.60 ± 1.80 | 15.93 ± 0.80 | 14.05 ± 1.78 |
| TP content  (mg g⁻¹) | 2.37 ± 1.12 | 1.94 ± 0.32 | 2.79 ± 0.48 | 1.78 ± 0.15 |
| N/P ratio | 8.78 ± 3.78 | 8.62 ± 0.52 | 5.82 ± 1.01 | 7.93 ± 1.25 |

Table S5 Soil OC and nutrient contents (mean ± SD) at initial and final sampling in the two substrate treatments (n = 3).

| Parameters | Treatment PS | | Treatment S | |
| --- | --- | --- | --- | --- |
|  | Initial | Final | Initial | Final |
| OC content  (mg g⁻¹) | 3.09 ± 0.61 | 5.46 ± 1.32 | 0.36 ± 0.08 | 3.85 ± 0.10 |
| TN content  (mg g⁻¹) | 0.29 ± 0.1 | 0.63 ± 0.10 | 0.19 ± 0.04 | 0.47 ± 0.06 |
| TP content  (mg g⁻¹) | 0.17 ± 0.01 | 0.15 ± 0.03 | 0.17 ± 0.01 | 0.15 ± 0.01 |
| Soil NH₄⁺–N (µg g⁻¹) | 0.40 ± 0.11 | 0.47 ± 0.06 | 0.50 ± 0.14 | 0.46 ± 0.04 |
